# Delay Along the Care Seeking Journey of Patients with Microbial Keratitis in Uganda

**DOI:** 10.1080/09286586.2019.1616775

**Published:** 2019-05-15

**Authors:** Simon Arunga, Guyguy M. Kintoki, Stephen Gichuhi, John Onyango, Rob Newton, Astrid Leck, David Macleod, Victor H. Hu, Matthew J. Burton

**Affiliations:** a International Centre for Eye Health, London School of Hygiene & Tropical Medicine, London, UK; b Department of Ophthalmology, Mbarara University of Science and Technology, Mbarara, Uganda; c Department of Ophthalmology, University of Nairobi, Nairobi, Kenya; d Department of Ophthalmology, Uganda Virus Research Institute, Entebbe, Uganda; e Tropical Epidemiology Group, London School of Hygiene & Tropical Medicine, London, UK

**Keywords:** Microbial keratitis, bacterial keratitis, fungal keratitis, keratitis, blindness, Uganda

## Abstract

**Purpose:**

To describe the care seeking journey and causes of delay among patients with Microbial Keratitis in Uganda.

**Methods:**

A prospective cohort of patients presenting with microbial keratitis at the two main eye units in Southern Uganda (2016–2018). We collected information on demographics, home address, clinical history, and presentation pathway including, order of facilities where patients went to seek care, treatment advice, cost of care, and use of Traditional Eye Medicine. Presentation time was noted. We compared “direct” presenters versus “indirect” presenters and analysed predictors of delay.

**Results:**

About 313 patients were enrolled. All were self-referred. Only 19% of the patients presented directly to the eye hospital. Majority (52%) visited one facility before presenting, 19% visited two facilities, 9% visited three facilities, and 2% visited four facilities. The cost of care increased with increase in the number of facilities visited. People in a large household, further distance from the eye hospital and those who used Traditional Eye Medicine were less likely to come directly to the eye hospital. Visiting another facility prior to the eye hospital and use of Traditional Eye Medicine aOR 1.58 (95%CI 1.03–2.43), *p* = .038 were associated with delayed presentation to the eye hospital.

**Conclusion:**

This study provided information on patient journeys to seek care. Delay was largely attributable to having visited another health facility: a referral mechanism for microbial keratitis was non-existent. There is need to explore how these health system gaps can be strengthened.

## Introduction

Microbial keratitis (MK) can be caused by a range of pathogens, including bacteria, viruses, protozoa (e.g. acanthamoeba), and fungi (yeasts, moulds, and microsporidia). It is characterised by an acute or sub-acute onset of pain, conjunctival hyperaemia, and corneal ulceration with a stromal inflammatory cell infiltrate. MK frequently leads to sight-loss from dense corneal scarring, or even loss of the eye, especially when the infection is severe and/or appropriate treatment is delayed.^1^ MK is important because it is a leading causes of uniocular blindness worldwide.^2,3^

In Sub Saharan Africa, the incidence of MK has been suggested to be around 180/100,000/year.^4^ Bacterial (*staphylococcus, streptococcus* and *pseudomonas)* and fungal (*fusarium* and *aspergillus*) are the most common with an almost 50:50 proportion.^5–11^

In Low and Middle-Income Countries (LMIC), MK management is often more challenging because of late presentation, use of Traditional Eye Medicine (TEM), insufficient diagnostic support, lack of effective drugs and keratoplasty services.^11,12^

A critical step in effectively managing MK is ensuring that patients start appropriate treatment as early as possible. This is because once the infection is well established, there is little that can be done to change its course.^13^ It is believed that many MK start following corneal abrasions. Studies in Burma and Bhutan showed that if people with a simple corneal abrasion applied antibacterial or antifungal medication within the first 24–48 hours, there was full recovery without any infectious sequalae.^14,15^

Delayed presentation of patients is a key determinant of outcomes.^12^ Patients typically present at least two weeks after the onset of the first symptoms.^12^ There are a number of factors that could contribute to this delay such as: distance from the hospital, transportation costs, poverty, self-medication, and tortuous referral pathways through the health system.^16–18^ Prior visit to a non-specialist health facility has been implicated as a cause of delay in other eye conditions.^17,19^

In Uganda, the public health system has six levels, with the lowest point of care being at the village level (Village Health Committee).^20^ However, physically, a Health Centre II (HC II) is the lowest unit and is located at a parish level, HC III at sub-county level, HC IV at county level, district hospital (HC V), and referral hospital (HC VI). These units have quite different staffing and capacity in terms of service provision. There are several different levels of private health care providers as well. Patients are referred up this tier system depending on the complexity of their condition.

Therefore, to investigate the role of the health system in providing care and onward referral of people with MK, here we describe the presentation pathway and factors associated with delayed presentation, among patients with microbial keratitis in Uganda.

## Methods

### Ethical statement

This study followed the tenets of the Declaration of Helsinki. It was approved by the London School of Hygiene & Tropical Medicine Ethics Committee (Ref 10647), Mbarara University Research Ethics Committee (Ref 10/04–16) and Uganda National Council for Science and Technology (Ref HS-2303). Written informed consent in “*Runyankore*” the local language was obtained before enrolment. If the participant was unable to read, the information was read to them by the research assistant. The participant was then asked to place a thumbprint on the consent form which was independently witnessed.

### Study design and setting

This was part of a study where we prospectively enrolled patients with MK that presented to Ruharo Eye Centre (REC) and Mbarara University and Referral Hospital Eye Centre (MURHEC) from December 2016 to March 2018. MURHEC is a government owned tertiary eye unit established in 2013. It provides mostly free services and sees about 6,000–10,000 patients/year. REC is a church-based, fee-paying tertiary eye hospital founded in the 1960s. It sees about 20,000–25,000 patients/year. Both hospitals are in Mbarara Municipality, South-Western Region, Uganda, approximately 4 hours’ drive from Kampala. The two units are about 5 km apart and work closely together.

### Participants

All patients that were enrolled into the cohort study were included. In that cohort study, we aimed to recruit all MK cases presenting during a year in order to have a powerful sample set to answer detailed questions around the seasonal microbiological patterns. It was important to recruit for a full year as MK had been shown in other parts of the world to have seasonal variations in its’ epidemiology.^21^

### Study participants

The inclusion criteria for the bigger prospective study was the presence of acute MK at presentation to the hospital defined as EITHER (i) corneal epithelial ulceration (≥1 mm diameter) AND corneal stromal infiltrate AND evidence of acute ocular inflammation (e.g. Conjunctival injection/anterior chamber inflammatory cells/hypopyon); OR (ii) a corneal abscess (≥1 mm diameter) AND evidence of acute ocular inflammation. We excluded those not willing to participate, those not willing to return for follow-up, pregnant women, lactating mothers, those aged below 18 years.

### Data collection procedures

Patients presenting with MK were introduced to the study and the informed consent processes followed. They were assigned a unique study number and their age, sex, occupation, and place of residence recorded. A history was taken of the circumstances in which their eye became infected, the predisposing factors (such as trauma and use of Traditional Eye Medicine [TEM]). A meticulous “journey” history was taken to document the date when they developed symptoms, where and when they sought treatment (name and level of the health centre), what medical advice and treatment was given (including whether they were referred to the eye hospital or not), how much each step cost them in Uganda shillings (transportation, consultation fees, medicines). The total amount of money recorded was for all the costs incurred before patients were enrolled into the study.

The place where they first received any form of treatment was denoted as “Facility 1”, the second place visited (either as a result of formal referral or self-initiated referral) was denoted “Facility 2” and so on. GPS coordinates were generated for the patients’ addresses (to the nearest village, parish, county school, or health centre depending on what was available on Google maps). Presenting Log MAR (Logarithm of Minimum Angle of Resolution) visual acuity at 2 m in a dark room was measured using Peek Acuity software.^22^ For visual acuities of counting fingers or less, Log MAR values were attributed as follows: counting fingers, 2.0; hand movements, 2.5; perception of light, 3.0; and no perception of light, 4.0.^23^ The patients were then examined on a slit lamp and clinical signs carefully recorded. Infiltrate size was measured as the greatest diameter of the infiltrate (dimension 1) and the diameter of an imaginary line perpendicular to the widest axis (dimension 2). The final infiltrate size was then derived as the geometrical mean of the two diameters.^24^ The same was repeated after fluorescein staining of the ulcer to measure the epithelial defect sizes. Corneal specimens were obtained for microbiological testing at Mbarara University Microbiology Department. Patients were treated as per the hospital treatment protocol and followed up periodically for up to 3 months to determine their outcome.

### Analysis

Data were analysed in STATA v14. “direct” presenters were defined as participants whose first point of care was the eye hospital (MURHEC or REC). “Indirect” presenters are those who first went to other health centres before presenting to the eye hospital. Summary frequency tables of demographics and clinical presentation of “direct” versus “indirect” presenters were generated with appropriate statistical tests for each variable (Wilcoxon rank sum for the continuous variables and χ^2^ test for the categorical variables). To determine where the participants came from, Google maps was used to pinpoint to the addresses of the participants. The presentation journey was described using interval times in days from home to Facility 1 or from Facility 1 to Facility 2 and so on (presented as median time in days with Inter Quartile Ranges [IQRs]). To describe the cost of care, the total patient expenditure at different facilities were summarised and cumulative expenditure derived depending on how many facilities an individual visited. Costs are presented as median expenditure in Uganda shillings with IQRs.

Presentation time was defined as the time in days it took a patient to come to the eye hospital after onset of symptoms. For analysis of delay, presentation time was divided into quartiles as “early” (0–7 days), “intermediate” (8–14 days), “late “(15–30 days), and “very late” (>30 days). Ordinal logistic regression was performed to determine the factors associated with these four quartiles of “delay”, while logistic regression was performed to determine factors associated with direct presentation. Univariable regression was performed to generate crude Odds Ratios (OR). After assessing for collinearity, variables with a *p* value less than 0.1 were introduced in the multivariable model. A backward stepwise approach was then used, until only the variables with a *p* value <0.05 were retained. Adjusted OR were reported for the final model.

## Results

### Demographic features

During the study period, 313 patients were enrolled into this study. The baseline characteristics of direct versus direct presenters are shown in Table 1. Overall, the direct and indirect presenters were similar for many variables. However, the direct presenters lived closer to the eye hospital (median 58 km vs. 87 km; *p* = .0001), had fewer household members (median 5 people vs. 7 people; *p* = .006) and fewer were farmers (59% vs. 73%, *p* = .031).

**Table 1.** Baseline characteristics of direct versus indirect presenters (*n* = 313).

	Direct presenters (*n* = 58)			Indirect presenters (*n* = 255)			
Variable	Median	(IQR)	(Total range)	Median	(IQR)	(Total range)	*p* value
Age	47	(35–60)	(18–96)	47	(35–60)	(18–87)	0.772
Distance to eye units	58	(16–85)	(0.2–244)	87	(57–131)	(2–378)	0.0001
Household population	5	(3–7)	(1–14)	7	(4–8)	(1–28)	0.006
Distance to nearest health centre in km*	2	(1–3)	(0–14)	3	(1–4)	(0–45)	0.174
Variable	Category	Count	(%)	Count	(%)		*p* value
Gender	Female	22	(38%)	117	(46%)		0.271
	Male	36	(62%)	138	(54%)		
Occupation	Farmer	34	(59%)	186	(73%)		0.031
	Nonfarmer	24	(41%)	69	(27%)		
Marital status	Unmarried Ɨ	18	(31%)	77	(30%)		0.900
	Married	40	(69%)	178	(70%)		
Education status	None	15	(26%)	69	(27%)		0.407
	Primary	29	(50%)	133	(52%)		
	Secondary	7	(12%)	38	(15%)		
	Tertiary	7	(12%)	15	(6%)		
Being head of household	Yes	42	(72%)	170	(67%)		0.398
	No	16	(28%)	85	(33%)		
Needed an escort to hospital*	Yes	24	(41%)	49	(20)		<0.0001
	No	34	(59%)	202	(80)		

*Variables with some missing data: distance to nearest health centre was measured in km (*n* = 312, [direct 57]) needed an escort (*n* = 309, [direct 58]). Ɨ Unmarried included single, divorced, and widowed,

Table 2 shows some select clinical history and signs of direct versus indirect presenters. Compared to indirect presenters, direct presenters had a shorter presentation time (median 8 days vs. 17 days; *p* < .0001), had slightly better presenting vision (median Log MAR 0.65 vs. 1.3; *p* = .075), a smaller infiltrate size (median 4.2 mm vs. 5.5 mm; *p* = .025) and a smaller epithelial defect (median 3.5 mm vs. 4.1 mm; *p* = .048). The proportion of people who had used TEM was higher among the indirect (63%) versus direct presenters (46%), *p* = .020. The direct and indirect presenters had similar proportions with a history of trauma, hypopyon, an opaque stromal opacity and perforation.

**Table 2.** Clinical history and clinical signs of direct versus indirect presenters (*n* = 313).

	Direct presenters (*n* = 58)			Indirect presenters (*n* = 255)			
Variable	Median	(IQR)	(Total range)	Median	(IQR)	(Total range)	*p* value
Presentation time in days*	8	(2–18)	(0–116)	17	(8–32)	(0–370)	<0.0001
Presenting vision (Log MAR)	0.65	(0.1–2.5)	(0–4)	1.3	(0.3–2.5)	(0–4)	0.072
Infiltrate size in mm Ɨ	4.2	(2.5–7.1)	(0.9–11)	5.5	(3.5–8)	(0.5–13)	0.025
Epithelial defect size in mm Ɨ	3.5	(1.8–5.8)	(0–11)	4.1	(2.5–6.9)	(0–13)	0.048
Variable	Category	Count	(%)	Count	(%)		*p* value
History of trauma (overall 29%) ǂ	Yes	14	(25%)	77	(30)		0.388
	No	43	(75)	177	(70)		
Used traditional eye medicine (overall 61%)	Yes	27	(46)	161	(63)		0.020
	No	31	(53)	94	(37)		
Pain being the main complaint	Yes	26	(45%)	112	44		0.121
	No	32	55	143	56		
Opaque stromal opacity ǂ	Yes	25	(43)	107	(44)		0.918
	No	33	(57)	137	(56)		
Hypopyon ǂ	Yes	13	(22)	81	(32)		0.151
	No	45	(78)	172	(68)		
Perforated at admission	Yes	10	(17)	66	(26)		0.166
	No	48	(83)	189	(74)		

*Presentation time was measured as duration in days it took to come to the eye hospital after onset of symptoms. Ɨ geometrical of the largest diameter and the diameter perpendicular to the largest diameter. ǂ variables that had less than 313 observations due to missing data (trauma *n* = 311 [direct57], opaque stromal opacity *n* = 302 [direct 58], hypopyon *n* = 311 [direct 58]).

### Factors associated with direct presentation

On univariable and multivariable analysis summarised in Table 3. People who lived far from the eye hospital (overall *p* = .003), those from large households OR 0.53 (95%CI 0.32–0.85), *p* = .0080 and those who had used TEM OR 0.48 (95% CI 0.25–0.90), *p* = .020 were less likely to be direct presenters.

**Table 3.** Univariable and multivariable logistic regression analysis of factors associated with direct presentation to the eye hospital (*n* = 309).

Variable	Univariable analysis			Multivariable analysis		
	cOR	(95% CI)	*p* value	aOR	(95% CI)	*p* value
**Age in years**	1.004	(0.987–1.022)	0.576			
**Sex (being male)**	1.38	(0.77–2.48)	0.273			
**Marital status (being married)**	0.96	(0.52–1.78)	0.900			
**Occupation (being a farmer)**	0.52	(0.29–0.94)	0.033			
**Being head of household**	1.31	(0.69–2.46)	0.399			
**Number of people in household** (increase/one person)	0.59	(0.38–0.90)	0.015	0.53	(0.32–0.85)	0.008
**Distance to the eye hospital**						
0–50 km	1		0.001			0.003
50–100 km	0.52	(0.26–1.01)		0.62	(0.30–1.27)	
100–150 km	0.16	(0.05–0.44)		0.16	(0.06–0.48)	
>150 km	0.42	(0.17–1.03)		0.52	(0.19–1.34)	
**Distance from nearest health centre** (increase per 1 km)	0.92	(0.822–1.029)	0.146			
**Positive history of trauma**	0.74	(0.38–1.44)	0.389			
**Positive history of TEM Use**	0.50	(0.28–0.90)	0.021	0.48	(0.25–0.90)	0.020
**Education status**						
None	1		0.462			
Primary	1.00	(0.50-1.99)				
Secondary	0.84	(0.31–2.25)				
Tertiary	2.14	(0.74–6.17)				

*patients with missing data were dropped from the model. OR less than 1 means they were less likely to come directly to the eye hospital

### Care seeking pathway

**Figure 1.**
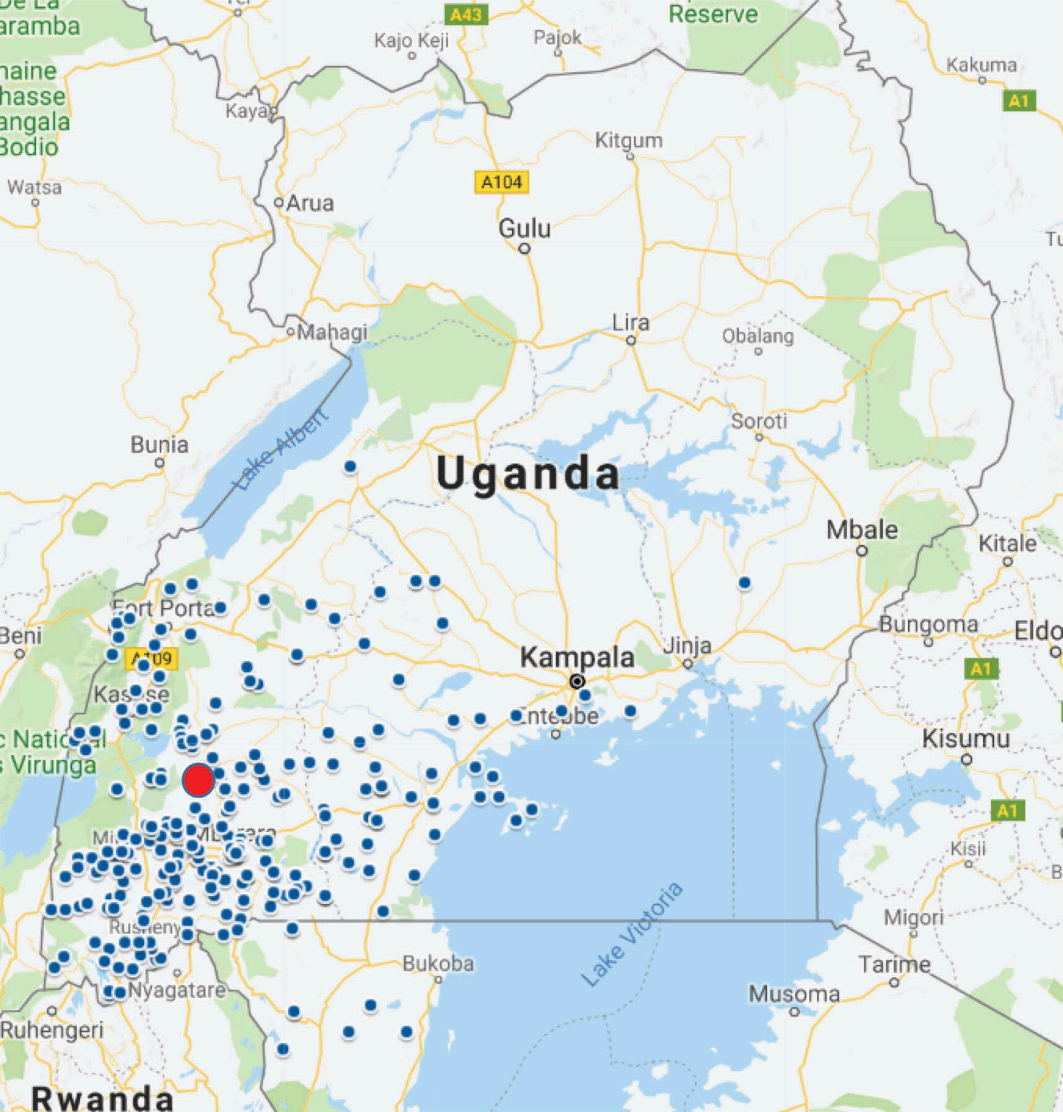
A map of Uganda showing patients homes.

**Figure 2.**
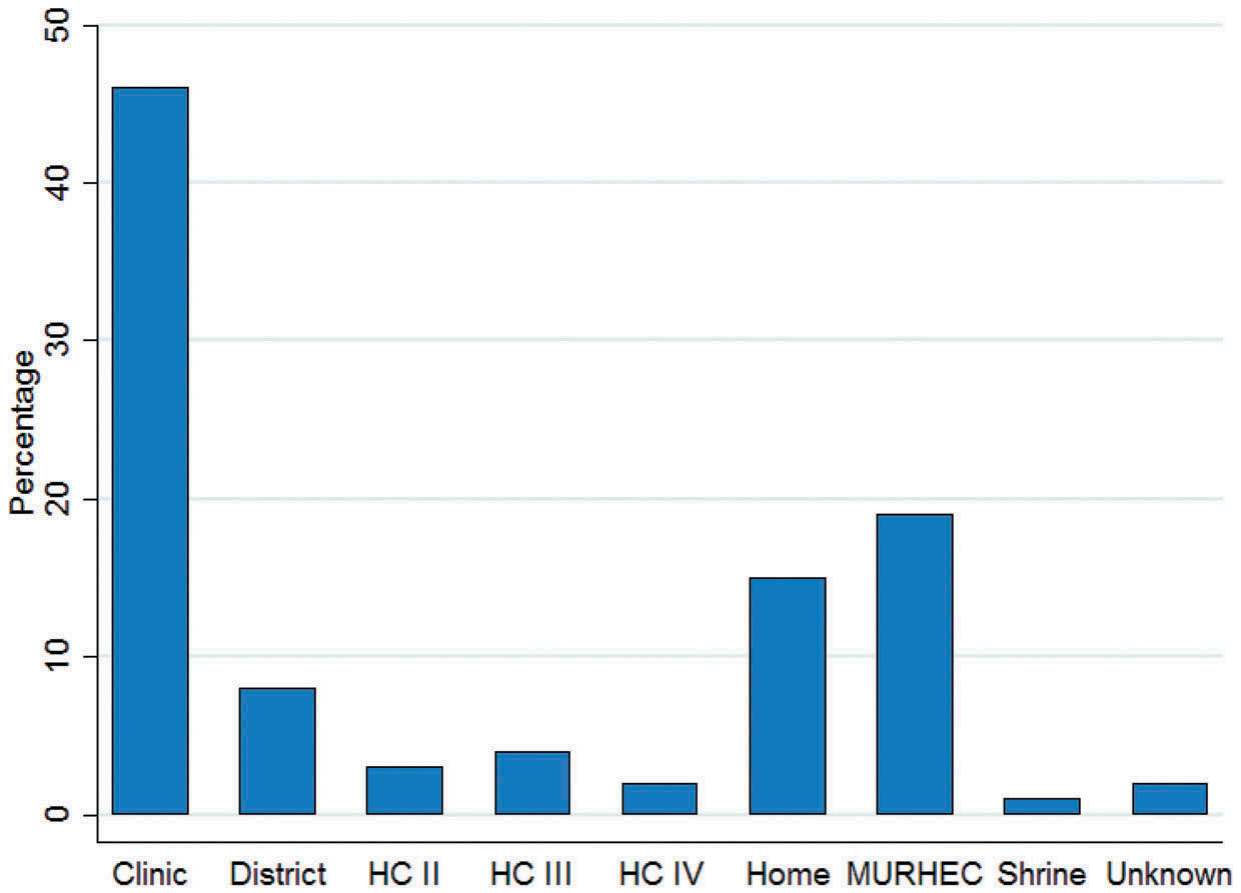
showing where patients first accessed treatment (*n* = 309).

Figure 1 shows where the patients came from in relation to the eye hospital (MURHEC or REC). Most came from the South Western region of Uganda and a handful from Northern Tanzania. Figure 2 shows the place where patients were first treated. Majority (46%) sought treatment at a nearby clinic/pharmacy/drug shop, 19% presented directly to the eye hospital, 15% were initially treated at home (either used TEM or an old eye drop) and 17% were treated at various levels of the health system (HC II, HC III, HC IV, and district hospital). Some patients (2%) did not know the type of facility where they first sought care and only 1% went to a traditional healer’s shrine for treatment.

**Figure 3.**
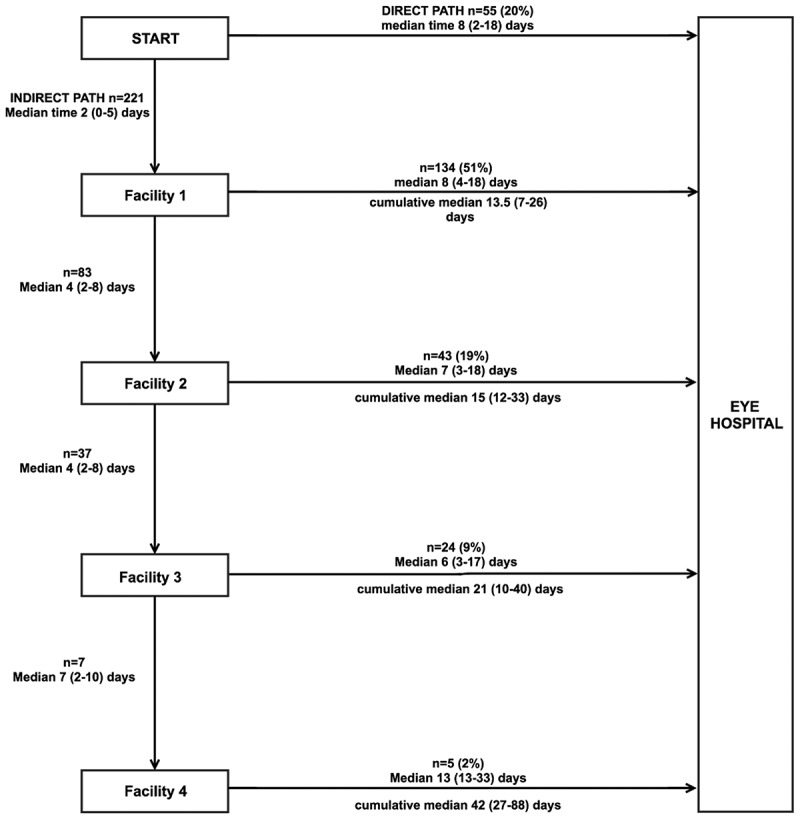
The care seeking journey of patients with microbial keratitis and the time taken at each step (*n* = 276).

Figure 3 illustrates the pathway patients took to come to the eye hospital and the different times spent on each stage. Only 55 (20%) patients presented directly to the eye hospital, majority (134, 51%) visited one facility before presenting to the eye hospital, another 43 (19%) visited two facilities, 24 (9%) visited three facilities, and 5 (2%) visited four facilities. On average, patients took about a week to move from one facility to the next. The shortest response time was from onset of symptoms to Facility 1 and was even shorter among indirect presenters, median 2 days (IQR 0–5) versus direct presenters, median 8 (IQR 2–18), *p* < .0001. The longest interval time was from Facility 4 to the eye hospital, median 13 (IQR 10–33). The choice of the first facility did not affect overall presentation time. All the patients were self-referred.

We found in our study that most patients used TEM after having been to a health facility (secondary use). Out of the 188 who used TEM, only 51 used TEM as primary treatment (47 at home and 4 at the traditional healers’ shrine). The rest (137/188) had secondary TEM application.

### Cost of care

The cost of care in Uganda shillings (UGX) is presented in Table 4. The cost of care increased with increase in the number of facilities visited. There was evidence (Cuzick test for trend *p* < .0001), of an association between expenditure and number of facilities visited prior to presentation. The lowest spend was for direct presenters where the median expenditure was UGX 30,000 (IQR 7,000–63,000, total range 0–385,000) and the largest spend was among patients who had visited 4 facilities before presentation with a median expenditure of UGX 284,000 (IQR 118,000–439,500, total range 96,000–864,000). Across the different expenditure lines, medicines were the most expensive followed by transportation, consultation fees were the least expensive.

**Table 4.** Money spent by patients per number of facilities visited before coming to the eye hospital.

			Cost of care median (IQR) in Uganda Shillings*							
Facility	*n*	(%)	Transportation		Consultation		Medicine		Total expenditure	
0	58	(18.5%)	11,000	(4,000–20,000)	15,000	(0–15,000)	0	(0–27,000)	30,000	(7,000–63,000)
1	147	(52%)	19,500	(10,000–33,000)	15,000	(15,000–15,000)	19,800	(2,750–99,500)	52,000	(31,000–142,000)
2	58	(18.5%)	22,000	(15,000–37,000)	15,000	(0–15,000)	25,750	(6,000–80,000)	67,750	(34,250–142,500)
3	29	(9%)	30,000	(19,000–51,000)	15,000	(0-15,000)	28,500	(3,000–70,000)	78,250	(32,000–209,000)
4	6	(2%)	62,500	(33,000–143,000)	12,500	(10,000–30,000)	170,500	(78,000–343,500)	284,000	(118,000–439,500)
*p* value of test for trend									<0.0001	

*All money is quoted in Uganda shillings. The US $ exchange rate was US $1: Uganda shillings 3,700 (2017). Ɨ0-direct presenters who did not visit any other facility before coming to the eye hospital. Patients with incomplete data were not included in this analysis

### Factors associated with delay

We tested for associations with delay in presenting to the eye hospital (Table 5). After adjusting for distance, visiting another facility prior to the eye hospital was strongly associated with delay but no obvious trend. Previous use of TEM was also found to be associated with delay OR 1.58 (IQR 1.03–2.43), *p* = .038

**Table 5.** Univariable and multivariable ordinal logistic regression analysis of factors associated with delay among patients with microbial keratitis (*n* = 309).

Variable	Univariable analysis			Multivariable analysis		
	cOR	(95% CI)	*p* value	aOR	(95% CI)	*p* value
**Age in years**	1.009	(0.994–1.019)	0.140			
**Sex (being male)**	1.06	(0.71–1.58)	0.792			
**Marital status (being married)**	0.86	(0.55–1.33)	0.316			
**Occupation (being a farmer)**	1.24	(0.80–1.93)	0.339			
**Being head of household**	0.83	(0.54–1.27)	0.394			
**Number of people in household** (increase/one person)	1.14	(0.85–1.51)	0.365			
**Distance to the eye hospital** (every 10km increase)	1.036	(1.003–1.)	0.034			
**Distance from nearest health centre** (increase per 1km)	1.01	(0.97–1.06)	0.501			
**Positive history of trauma**	0.96	(0.62–1.49)	0.860			
**Positive history of TEM Use**	1.73	(1.14–2.62)	0.010	1.58	(1.03–2.43)	0.038
**Other facilities visited before eye hospital**						
Nil (direct presenters)	1		0.0002	1		0.001
One facility	2.95	(1.63–5.38)		2.74	(1.53–4.92)	
Two facilities	3.62	(1.74–7.52)		2.58	(1.30–5.15)	
Three facilities	4.12	(1.82–9.34)		3.26	(1.42–7.45)	
Four facilities*	15.5	(2.65–90)		14.3	(2.45–83.7)	

*two patients had visited five facilities and one patient six facilities, these were dropped from the analysis

## Discussion

This study aimed to describe the presentation journey and factors associated with delay. Factors associated with delay were having visited another health facility and prior use of Traditional Eye Medicine (TEM). This supported our hypothesis that an initial visit to a health facility introduced delay as had been reported previously for other eye conditions.^17,19,25^ After onset of symptoms, the majority of patients quickly visited a health facility to seek treatment. This was an impressive median response time (within 48 hours). Although we did not explicitly ask their reasons for presenting early to these facilities, the painful nature of MK, proximity of the facilities and trauma (for those who had it) could have played a role. Perhaps, if appropriate treatment had been given or rapid referral made at this stage, the outcomes might have been better.^13,14^

At the first point of contact with the health system, there were three missed opportunities that we identified in our study, these were: to promptly initiate appropriate treatment; to triage and urgently refer; and health education advice against TEM use. We discuss these below.

Firstly, the health facility where most patients presented first were usually a nearby pharmacy/clinic. These are mostly private clinics that have sprouted up in many parts of Uganda. They are loosely regulated, manned by primary health workers and do not require a doctor’s prescription to dispense treatment. Effective anti-microbial medication such as Natamycin and Ciprofloxacin eye drops are not available in such units. These could be potential stakeholders to target in promotion of triage and referral mechanisms for MK. We found that there was no referral mechanism for MK: all patients who came to the eye hospital were self-referred.

Secondly, all the patients who visited a health facility we given some treatment but none of the patients was ever referred for specialist care. Most of the health centres (II and III) are managed by mid-level cadres, who may not have the necessary skills and tools to appreciate the urgency and seriousness of MK. General eye health training has been previously reported to be limited among mid-level cadres in the region.^26^ In addition, Uganda is still grappling with a major shortage of human resources for eye health. An eye specialist is found at some level six facilities and a mid-level ophthalmic cadre might be available in some level IV onwards.^27^ We plan to conduct a study into factors around the health system that could be developed to strengthen treatment, triage and referral.

Thirdly, we found in our study that most patients used TEM after having been to a health facility (secondary use). This is worrying because these were patients who could have been sensitised against TEM use at the health facilities where they first presented. This was a missed opportunity that needs to be addressed.

Fifty-eight (19%) of the patients were direct presenters. As expected, people who had large households, those who lived far from the eye hospital and those who used TEM were less likely to present directly to the eye hospital. Understandably, use of TEM and having a large household were negative predictors for being a direct presenter. Most of the people who used TEM used it at home and this was marked as a treatment event in our study design. Many patients in our cohort were heads of households and the sole bread winners, they might have preferred to first seek treatment at a place near home.

The cost of care was variable depending on the number of facilities visited. Most of the money was spent on drugs, and transportation. The public health system in Uganda is largely free or highly subsided. Expenses are incurred on transportation and sometimes medicines when they are out of stock. For the case of MK, drugs such as Natamycin have only been erratically and expensively supplied by select private pharmacies and not available in the public health system. We anticipate this to change as Natamycin was recently added on the WHO essential medicines list.^28^

### Strengths/limitations

This study was the first in SSA to systematically collect information on how MK patients seek care and what influences their pattern. It provides useful information on key health system gaps that need strengthening. Before this study, it had been thought that patients had poor health seeking behaviour, however, what we found was that majority of people presented to a health facility quite early after the onset of symptoms. Secondly, although TEM use was a known problem, this study showed that the bigger problem was secondary TEM use, that is patients who opted to use TEM even after they had been to a health facility.

Although we collected information on distance covered and treatment given at each level, it was difficult to analyse for these because most patients did not come to the eye hospital with their medicine and could not recall the names. There were many circular movements that made it complicated to analyse total distance covered by each patient. A qualitative approach in discussing with patients what informed their choice of self-referral or direct presentation would have strengthened the evidence in this study.

## Conclusion

Delayed presentation to a specialist eye hospital is a problem in the care of MK, and that this appears to be largely attributable to slow referral through the health system. There are opportunities for health education, early referral, appropriate treatment and sensitization against TEM use that could be utilized to improve care of MK. More needs to be done to understand what goes on in the health system and how this can be strengthened.

## Submission statement

This submission has not been published anywhere previously and that it is not simultaneously being considered for any other publication.
